# Co-doping of Ag into Mn:ZnSe Quantum Dots: Giving Optical Filtering effect with Improved Monochromaticity

**DOI:** 10.1038/srep14817

**Published:** 2015-10-08

**Authors:** Zhiyang Hu, Shuhong Xu, Xiaojing Xu, Zhaochong Wang, Zhuyuan Wang, Chunlei Wang, Yiping Cui

**Affiliations:** 1 Advanced Photonics Center, School of Electronic Science and Engineering, Southeast University, Nanjing, 210096 (P. R. China)

## Abstract

In optics, when polychromatic light is filtered by an optical filter, the monochromaticity of the light can be improved. In this work, we reported that Ag dopant atoms could be used as an optical filter for nanosized Mn:ZnSe quantum dots (QDs). If no Ag doping, aqueous Mn:ZnSe QDs have low monochromaticity due to coexisting of strong ZnSe band gap emission, ZnSe trap emission, and Mn dopant emission. After doping of Ag into QDs, ZnSe band gap and ZnSe trap emissions can be filtered, leaving only Mn dopant emission with improved monochromaticity. The mechanism for the optical filtering effect of Ag was investigated. The results indicate that the doping of Ag will introduce a new faster deactivation process from ZnSe conduction band to Ag energy level, leading to less electrons deactived via ZnSe band gap emission and ZnSe trap emission. As a result, only Mn dopant emission is left.

In the past two decades, colloidal chemistry methods become the major way for synthesizing highly luminescent QDs in a liquid medium. Colloidal chemistry methods can either synthesize QDs in organic solution via an organometallic route or in water by an aqueous route^1^,^2^,^3^,^4^. Though only limited sorts of QDs can be synthesized in water, aqueous route exactly has many advantages. Water is environmentally-friendliness and is able to dramatically decrease the synthesis cost. QDs prepared by aqueous route have good bio-compatibility, low synthesis temperature below 100 °C, high synthesis reproducibility, and the ability for scaling up synthesis. All these advantages make aqueous route with better industrial and commercial prospect^5^,^6^.

Because of the low toxicity and strong luminescence, Mn doped ZnSe QDs have been regarded as a promising substitution for Cd-contained QDs which are the workhorse in QD family in the recent two decades^7^,^8^,^9^,^10^. Presently, high quality Mn:ZnSe QDs with pure Mn emission were synthesized via an organometallic route. By ligand exchange and ZnS shell caption, the resulted QDs can be transferred into aqueous solution for bio-imaging application^11^,^12^. In comparison, though direct synthesis of Mn doped ZnSe QDs by the aqueous route is a more simple way, as-prepared aqueous Mn:ZnSe QDs still suffer from low monochromaticity and low photoluminescence (PL) quantum yield (QY)^13^,^14^. One major problem is the strong ZnSe trap emission accompanied with Mn dopant emission. ZnSe trap emission originates from defects on either QD surface or QD inside (especially the interface between impurity and QD host). Surface defects-induced trap emission can be eliminated by improved surface ligand modification^15^ or shell capping^16^. As for internal defects-induced trap emission, there is still no effective solution^17^,^18^,^19^,^20^.

In this work, we showed that the low monochromaticity of aqueous Mn:ZnSe QDs can be improved by co-doping of non-irradiative Ag inside QDs. For the first time, we observed an optical filtering effect in nanosized QDs. Namely the doping of Ag can filter the emissions from ZnSe band gap and ZnSe trap, but allow the transmission of Mn dopant emission. Hence, aqueous doped ZnSe QDs with improved monochromaticity can be achieved. As far as we know, previous works about co-doped ZnSe mainly focused on Cu and Mn co-doped ZnSe QDs with white light emission. In that case, both Cu and Mn dopants act as irradiative centers^19^,^21^, and hence no optical filtering effect is observed. The current work is the first report to improve the monochromaticity of QDs by using an optical filtering effect in nanosized QDs.

## Results

### Synthesis and characterization of Mn:ZnSe QDs and Ag,Mn:ZnSe QDs

**Figure 1:**
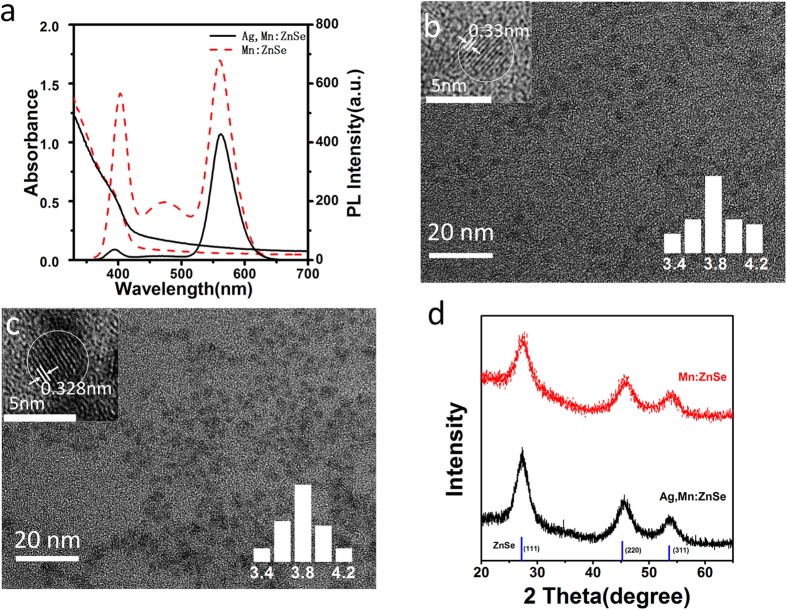
Optical properties and characterization of QDs. (**a**) Absorption and PL spectra of Mn: ZnSe and Ag,Mn:ZnSe QDs. (**b**) TEM, HRTEM images and size distributions (inset) of Mn:ZnSe QDs. (**c**) TEM, HRTEM images and size distributions (inset) of Ag,Mn:ZnSe QDs. (**d**) XRD patterns for Mn:ZnSe QDs and Ag,Mn:ZnSe QDs.

In this paper, Ag and Mn co-doped ZnSe (namely Ag,Mn:ZnSe) QDs and Mn doped ZnSe (namely Mn:ZnSe) QDs were prepared by a nucleation doping strategy (see Methods section). Figure 1a shows the absorption and PL spectra of Mn:ZnSe QDs and Ag,Mn:ZnSe QDs synthesized at the same experiment conditions. From PL spectra, Mn:ZnSe QDs possess three emission bands: namely ZnSe band gap emission around 400 nm, ZnSe trap emission around 480 nm, and Mn dopant emission around 580 nm. After co-doping of Ag however, Ag,Mn:ZnSe QDs show only pure Mn dopant emission. The ZnSe band gap emission and ZnSe trap emission disappear after co-doping of Ag. The PL QY of Mn dopant emission is 13.1% for Ag,Mn:ZnSe QDs, similar to that of Mn dopant emission (17.1%) in Mn:ZnSe QDs. Apparently, PL results indicate that Ag impurity can filter ZnSe band gap emission and ZnSe trap emission but less affect Mn dopant emission. Note that a slight blueshift in ZnSe band gap emission is observed after Ag doping, which is caused by the different growth rate and size of ZnSe after Ag doping.

From absorption spectra, the main difference between two types of QDs is a slightly elevated absorption baseline from 430 nm to 600 nm, which is caused by the doping of Ag as observed in the case of Ag:ZnSe QDs^22^,^23^. The size is calculated as 4.0 nm for Mn:ZnSe QDs and 4.1 nm for Ag,Mn:ZnSe QDs according to the position of the first excitonic peak^24^. Figure 1b,c shows transmission electron microscopy (TEM) images of Mn:ZnSe and Ag,Mn:ZnSe QDs respectively. As can be seen, the as-prepared Ag,Mn:ZnSe QDs are quasi-spheres with average size of 3.9 nm, which is in agreement with the size (3.9 nm) of Mn:ZnSe QDs. It suggests no serious change in QD size after doping of Ag into Mn:ZnSe QDs. The high-resolution (HR) TEM images indicate the distances between the adjacent lattice fringes are 0.33 nm for Mn:ZnSe QDs and 0.328 nm for Ag,Mn:ZnSe QDs, assigning to (111) facet of ZnSe crystals.

Figure 1d shows X-ray powder diffraction (XRD) patterns for Mn:ZnSe and Ag,Mn:ZnSe QDs. Both Mn:ZnSe and Ag,Mn:ZnSe QDs have three major peaks centered at 27.3, 45.4, and 53.7 degrees, which assign to the diffractions from (111), (220), and (311) facets of cubic zinc blend ZnSe. Obviously, the doping of Mn or Ag does not seriously change the crystal lattice of ZnSe QDs. Inductively coupled plasma atomic emission spectrometry (ICP) is used for measuring the composition of Mn:ZnSe QDs and Ag,Mn:ZnSe QDs. It should be emphasized that as-prepared QDs are purified before composition measurements. The excess free monomers in solution can be separated from QDs during the purification. As a result, the measured ICP results can reflect the real chemical composition of QDs. The measured ICP atomic ratio of Zn/Se/S/Mn is 39.3/26.4/13.9/1.0 for Mn:ZnSe QDs, whereas the ratio of Zn/Se/S/Mn/Ag is 41.1/30.1/12.9/1.0/1.76 for Ag,Mn:ZnSe QDs. Due to S atoms come from thiol ligands on QD surface, the similar atomic ratios of Zn/Se/S/Mn in Mn:ZnSe QDs and Ag,Mn:ZnSe QDs indicate their similar composition and thiol ligand modification. Since TEM results have suggested the similar size of these two QDs, aforementioned ICP results confirm that the doping of Ag can not seriously affect the growth of Mn:ZnSe QDs.

### Optimization of experimental conditions

In this work, the improved monochromaticity of QDs relies on experimental conditions during Ag co-doping. Since the poor monochromaticity of Mn:ZnSe QDs is induced by the multiple emission peaks, improved monochromaticity can be achieved by filtering two of these peaks. Herein we use the PL intensity ratio between Mn dopant emission at 580 nm and ZnSe band gap emission at 400 nm to describe the improved monochromaticity of QDs after Ag doping. Various experimental conditions have been found to affect QD monochromaticity by either suppressing ZnSe emission or improving Mn dopant emission.

**Figure 2:**
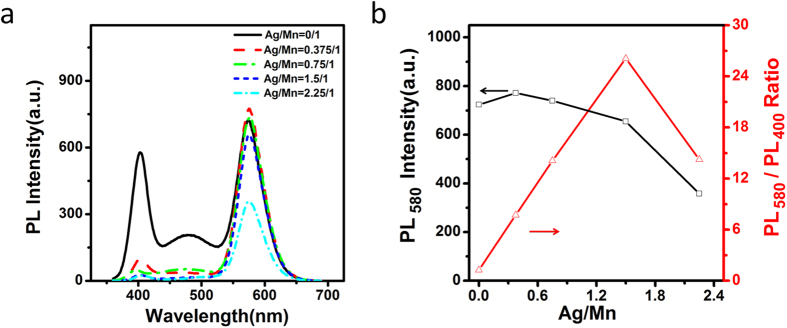
PL spectra (**a**) and the Mn emission peak intensity as well as the PL intensity ratios at 580 nm and 400 nm (**b**) of Ag,Mn:ZnSe QDs at different molar ratio of Ag/Mn.

The doping of Ag into Mn:ZnSe QDs is found able to suppress ZnSe emission, leading to improved QD monochromaticity. By keeping a constant Mn, Se and Zn concentrations, different amounts of Ag were doped inside Mn:ZnSe QDs. As can be seen from Fig. 2a, Mn dopant emission shows slight increase at Ag/Mn ratio below 0.375/1. Further increased Ag/Mn ratio to 1.5/1 leads to slightly decreased Mn dopant emission. In comparison, ZnSe band gap emission and ZnSe trap emission show a dramatically decrease even if a small Ag/Mn ratio of 0.375/1. Obviously, Ag dopants can effectively suppress to ZnSe band gap emission and ZnSe trap emission, but hardly affect Mn dopant emission. Figure 2b shows the PL intensity ratios between Mn dopant emission and ZnSe band gap emission. As can be seen, when Ag/Mn ratio is 1.5/1, the PL intensity ratio reaches the maximum. It means the best suppression effect of Ag to ZnSe band gap and ZnSe trap emission achieves at Ag/Mn doping amount of 1.5/1. In this work, Ag/Mn of 1.5/1 is set as the default doping amount of Ag if no specific illustration.

**Figure 3:**
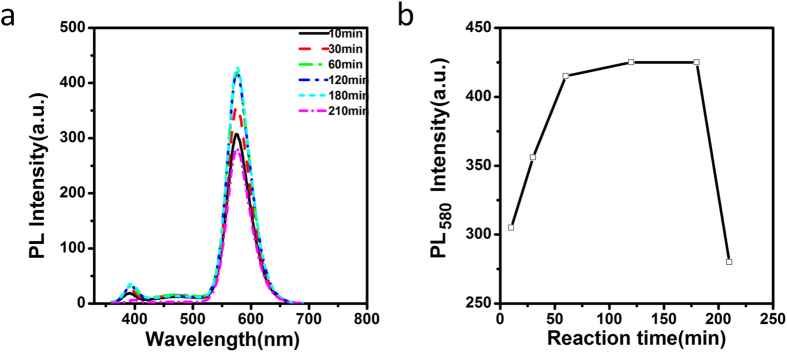
PL spectra (**a**) and Mn emission peak intensity (**b**) of Ag,Mn:ZnSe QDs at different reaction time.

Except Ag doping amount, the left conditions (growth time, MPA/Zn ratios and solution pH) mainly affect QD monochromaticity by changing Mn dopant emission. Figure 3 shows PL spectra of Ag,Mn:ZnSe QDs at different growth time after addition of Zn stock solution into the nuclei solution. With increased growth time below 60 min, Mn dopant emission increases dramatically. When the growth time is between 60 min and 180 min, Mn dopant emission almost keeps constant. Prolonged growth time leads to dramatic decrease for Mn dopant emission. Since ZnSe emissions change little during prolonged refluxing time, the changed monochromaticity of Ag,Mn:ZnSe QD at different growth time is mainly induced by the changed Mn emission.

**Figure 4:**
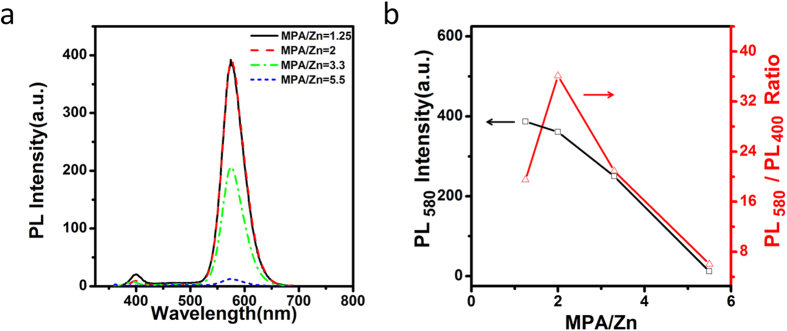
PL spectra (**a**) and the Mn emission peak intensity as well as the ratios of PL intensity at 580 nm and 400 nm (**b**) of Ag,Mn:ZnSe QDs at different molar ratio of MPA/Zn.

Figure 4 shows PL intensities of Ag,Mn:ZnSe QDs at different molar ratio of 3-Mercaptopropionic acid (MPA) ligand amount. When MPA/Zn ratios increase from 1.25 to 2, Mn dopant emission remains unchanged whereas ZnSe band gap and ZnSe trap emission decreased slightly, leading to improved suppression effect. When MPA/Zn ratios increase from 2 to 5.5, Mn dopant emission decreases dramatically, leading to decreased suppression effect. The optimal molar ratio of MPA/Zn is 2.

**Figure 5:**
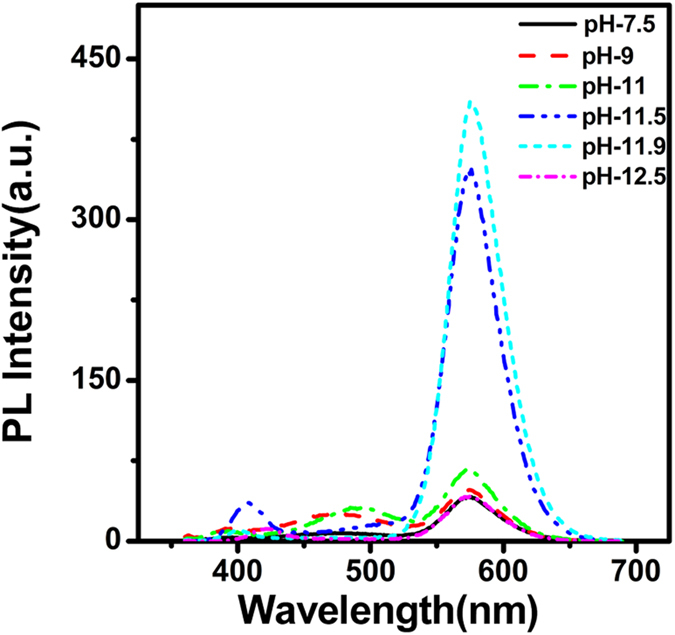
PL spectra of Ag,Mn:ZnSe QDs at different pH values.

The effect of solution pH on PL of Ag,Mn:ZnSe QDs is shown in Fig. 5. With increased pH values from 7.5 to 12.5, Mn dopant emission increases first and then decreases. The maximal PL of Mn impurity appears at pH 11.9. We think the appearance of an optimal pH is mainly determined by two opposite factors. (i) Since the logarithmic value of negative dissociation constant of thiol group on MPA ligand is 10.84, the present form of thiol changes from protonated group (RSH) to deprotonated (RS^−^) group during increased pH from 7.5 to 12.5. According to our previous report^25^, the protonation of thiol group at decreased pH promotes the detaching of ligand from QD surface, leading to decreased PL intensity at low pH. (ii) When the solution pH is extremely high (such as 12.5), Mn and Ag impurities tend to form precipitation of hydroxide, leading to decreased Mn dopant amount and emission.

In Fig. S1 in supporting information, the influence of growth time, MPA/Zn ratios and pH values on the emissions of Mn:ZnSe QDs was investigated. We can observe similar trend of Mn emission between Mn:ZnSe QDs and Ag,Mn:ZnSe QDs. It suggests that the improved monochromaticity of Ag,Mn:ZnSe QDs at the different conditions (growth time, MPA/Zn ratios and pH) is actually induced by the changed Mn emission. In comparison, Ag doping amount mainly affects the monochromaticity of Ag,Mn:ZnSe QDs via another way: namely suppressing ZnSe emission.

## Discussion

### Mechanism for Ag dopant-induced Optical Filtering

Aforementioned results have confirmed that the proper doping of Ag can filter ZnSe band gap and ZnSe trap emission but not filter Mn dopant emission. In the following text, we would like to discuss the possible effect of Ag during the recombination of excitons.

**Figure 6:**
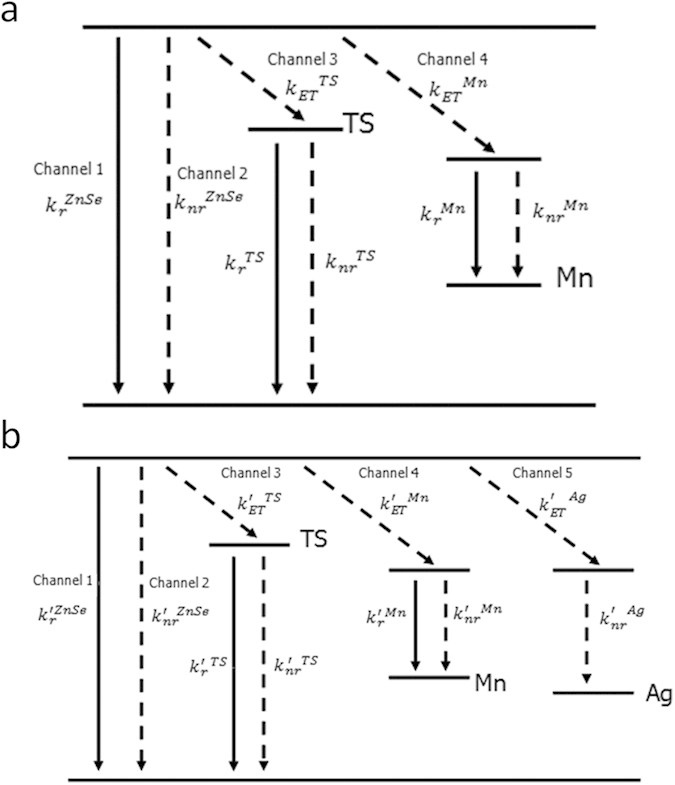
Energy levels illustration. Schematic illustration of energy levels of Mn:ZnSe QDs (**a**) and Ag,Mn:ZnSe QDs (**b**).

A schematic illustration for the energy levels for Ag,Mn:ZnSe QDs has shown in Fig. 6. Due to the use of nucleation doping strategy, the actually present state of Ag and Mn impurities are Ag_2_Se and MnSe inside QDs. According to the reference viewpoints^26^,^27^, the valence band and conduction band of Ag_2_Se and MnSe present inside the forbidden band of ZnSe QDs. The PL QY of ZnSe band gap emission for Mn:ZnSe (symbolized by *QY*_*ZnSe*_) and Ag.Mn:ZnSe QDs (symbolized by *QY’*_*ZnSe*_) can be expressed by:


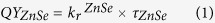



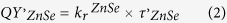


where 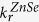 is the irradiative decay rates via ZnSe band gap emission. *τ*_*ZnSe*_ is the measured PL lifetimes of ZnSe band gap emission, which are shown in Fig. S2 in the supporting information. The single quote mark at the superscript indicates the displayed parameters coming from Ag,Mn:ZnSe QDs, whereas the parameters come from Mn:ZnSe QDs without quote mark.

The PL QYs of QDs can be measured from the emission of QDs. By dividing equation (1) using equation (2), the ratio of PL QYs with and without Ag doping is about 16. However, the measured PL lifetimes for ZnSe band gap emission in Mn:ZnSe and Ag,Mn:ZnSe QDs are similar as shown in Fig. S2 in the supporting information. It means the irradiative decay rates of ZnSe band gap emission in Mn:ZnSe QDs 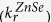 is about 16 times higher than that of Ag,Mn:ZnSe QDs 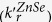. We also considered the influence of errors during PL QY and PL lifetime measurements. The results indicate that the errors could not lead to 16 times decrease of 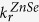 after doping Ag (see Supporting Information section). In other words, the co-doping of Ag into Mn:ZnSe QDs leads to decreased irradiative decay rates of ZnSe band gap emission 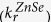. Similarly, according to the PL lifetimes and PL QYs, it can be also deduced that Ag doping can also decrease efficiency of electron transfer from ZnSe conduction band to defects energy level 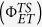 but cannot decrease the efficiency of electron transfer from ZnSe conduction band to Mn energy level 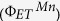 (see Supporting Information section).

**Figure 7:**
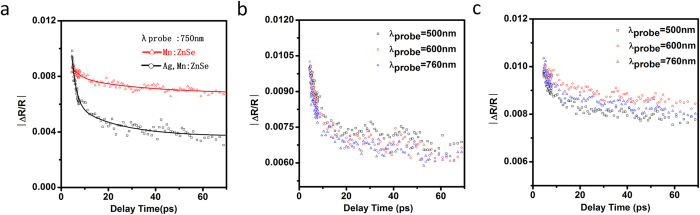
Transient absorption data of Mn:ZnSe and Ag,Mn:ZnSe QDs with 350 nm excitation and 750 nm probe (**a**). Transient absorption data for (**b**) Ag,Mn:ZnSe QDs and (**c**) Mn:ZnSe QDs with pump wavelength 350 nm and probe at 500–760 nm.

According to these results, we can summarize the effect of Ag by comparison of Mn:ZnSe QDs and Ag,Mn:ZnSe QDs. For Mn:ZnSe QDs without Ag, the electrons on ZnSe conduction bands would be consumed by three channels: namely recombination with holes on ZnSe valence band (channel 1), transferring to ZnSe trap energy level (channel 2), and transferring to Mn energy level (channel 3). This is also the reason why Mn:ZnSe QDs have three emission bands. After Ag doping inside Ag,Mn:ZnSe QDs in comparison, the electrons on ZnSe conduction bands are mainly consumed by two channels: namely transferring to Mn energy level (channel 4) and transferring to Ag energy level (channel 5). The electrons on Ag energy levels diminish via non-irradiation manner since no Ag dopant emission is observed in Ag,Mn:ZnSe QDs. There are two reasons supporting aforementioned mechanism. First, the doping of Ag lead to decreased 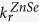 as mentioned above. It will lead to less electrons deactived via ZnSe band gap emission. Second, the doping of Ag introduce a new deactivation process from ZnSe conduction band to Ag energy level(channel 5 in Fig. 6). If channel 5 is a much faster deactivation process than channel 1 and 3, PL QY of ZnSe trap emission and ZnSe band gap emission will decrease. In Fig. 7, Pump probe transient absorption was used for monitoring exciton relaxation dynamics information with 350 nm excitation and 750 nm probe in Fig. 7. The fitted results (Table. S2 in Supporting Information) show an average decay time of 22.5 ps for the Mn:ZnSe QDs and 10.5 ps for Ag,Mn:ZnSe QDs. In comparison to Mn:ZnSe QDs, the decreased decay time for Ag,Mn:ZnSe QDs means the presence of much faster relaxation process^28^: namely channel 5 in Fig. 6. In addition, the data probed at 500–760 nm shows similar decaying feature in Fig. 7b,c, indicating the induced absorption signal is independent of probing wavelength in this spectral region. Obviously, transient absorption results confirm the presence of the second reason.

**Figure 8:**
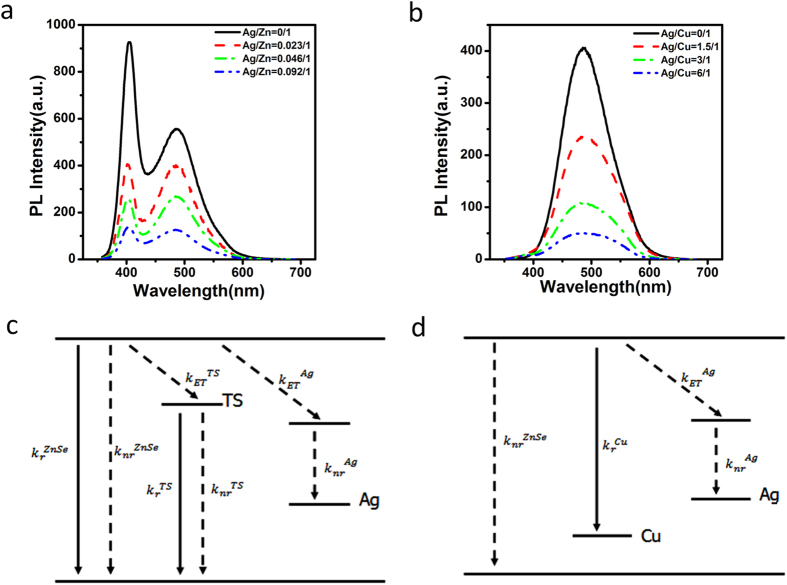
PL spectra of Ag:ZnSe QDs (**a**) and of Ag,Cu: ZnSe QDs (**b**) at different Ag doping amount. Schematic illustration of energy levels of Ag:ZnSe QDs (**c**) and Ag,Cu:ZnSe QDs (**d**).

To give more evidence about aforementioned mechanism, a contrast experiment was done as in Fig. 8. As can be seen, even if no Mn dopant inside QDs, the doping of Ag into pure ZnSe QDs can also lead to the quenched ZnSe band gap emission and ZnSe trap emission. It means that the observed optical filtering effect of Ag is independent of Mn impurity but relies on the interaction between Ag and ZnSe QDs. Furthermore, Cu:ZnSe QDs also show quenched emission after Ag doping (Fig. 8b). Since the emission of Cu:ZnSe QDs originates from ZnSe conduction band to Cu energy level (Fig. 8d), the quenched emission of Cu:ZnSe QDs suggests the interaction of Ag with ZnSe conduction band rather than with ZnSe valence band.

On the basis of above mechanism, the effect of various experimental conditions on the monochromaticity of QDs can be comprehended. Ag doping amount is a major condition affecting the monochromaticity. On one hand, Ag can suppress ZnSe band gap emission and ZnSe trap emission due to bringing a new energy transfer channel 5 in Fig. 6. Further increase of Ag doping amount can introduce more nonirradiated Ag centers inside QDs. It increases the energy transfer efficiency from QDs conduction band to Ag energy level^29^, leading to better suppression of ZnSe emission. This is the reason for the improved QD monochromaticity below Ag/Mn of 2.25. On the other hand, the doping of Ag also changes the local environment around Mn ions, leading to changed Mn emission^30^. However, this effect should only be taken in consideration only at extremely high Ag doping amount (Ag/Mn over than 2.25:1 in Fig. 2). Since the default Ag doping amount is Ag/Mn of 1.5/1 in this work, the influence of Ag to Mn emission can be neglected.

Other experimental conditions (growth time, MPA/Zn ratios and pH) affect QD monochromaticity mainly by changing Mn dopant emission. Only the optimal conditions (growth time, MPA/Zn ratios, and pH) can reduce defects during QDs growth process^31^,^32^. As a result, Mn dopant emission may be improved due to reduced nonirradiative recombination traps, whereas ZnSe trap emission may change little due to the offset of decreased irradiative recombination traps. In addition, the reduced defects at MnSe-ZnSe interface can also change the local environment around Mn ions, resulting in more efficient energy transfer from QDs to Mn and hence improved Mn emission. As a result, the PL intensity ratios between Mn dopant emission and ZnSe emission will be changed.

In conclusion, we synthesized Ag,Mn:ZnSe QDs in aqueous solution with improved monochromaticity. The co-doping of Ag can suppress ZnSe band gap and ZnSe trap emission but less affects Mn dopant emission. Under optimal conditions, ZnSe band gap and ZnSe trap emission can be fully suppressed by Ag, resulting in pure Mn dopant emission. Unlike to traditional Cu and Mn co-doped ZnSe QDs using two types of irradiative dopants, the current work doped non-irradiative Ag inside QDs, and thus observed optical filtering effect inside QDs for the first time. This work is also helpful to comprehend the coordination effect of two types of dopants inside a single QD.

## Methods

### Chemicals and materials

All materials used in this work were of analytical reagents. Zn(NO_3_)_2_, MnCl_2_, and AgNO_3_ were purchased from Beijing Chemical Factory. NaBH_4_ was purchased from Guangdong Chemical Reagent Engineering Technological Research and Development Center. NaOH was purchased from Shanghai Zhongshi Chemical Company. MPA and Se powder were purchased from Aldrich. NaHSe solution was prepared by using Se with NaBH_4_ according to the reference methods^33^.

### Apparatus

UV–vis absorption spectra were recorded with a Shimadzu 3600 UV–vis near-infrared spectrophotometer. PL measurements were performed with a Shimadzu RF-5301 PC spectrofluorimeter. The excitation wavelength was 350 nm with a 3/3 nm slit. PL decay results were measured by an Edinburgh FLS 920 spectrofluorimeter or Femtosecond Laser as excitation resource and oscilloscope as signal receiver. All optical measurements were performed at room temperature under ambient conditions. TEM images were recorded by a Tecnai F20 electron microscope with an acceleration voltage of 200 kV. XRD result was carried out by using the D/max-2500/PC. ICP results were measured by a PE5300DV ICP equipment. Purified QDs powder was used in ICP and XRD measurements. QD powder was obtained by precipitating QD from solution with equal volume of isopropanol and followed by drying in vacuum. We utilized a commercial Ti:sapphire regenerative amplifier (Libra, Coherent) at 800 nm (1.55 eV) with a repetition rate of 1 kHz and pulse duration of ~90 fs to carry out the TA experiments. For broadband measurements, a second-harmonic light source at 3.1 eV was used as the pump beam and an optical parametric amplifier (OperA solo, Coherent) was used to provide a probe beam with tunable wavelength. The relative polarizations of the pump and probe beams were set to be at the magic angle (54.7°).

### Preparation of Zn stock solution

The Zn(NO_3_)_2_ stock solution was prepared by dissolving 1.4875 g Zn(NO_3_)_2_·6H_2_O in 50 mL water (0.1 M), and followed by addition MPA. After then, the pH of Zn stock solution was adjusted to a specific value with the NaOH solution (5 M). The optimized molar ratio of MPA/Zn is 2 and the optimized pH value is 11.9.

### Synthesis of MnSe nuclei and Mn:ZnSe QDs

Nucleation doping strategy was used for synthesis Mn:ZnSe QDs^34^,^35^. Namely, MnSe nuclei were prepared first before growth of host ZnSe outside MnSe nuclei. Typically, the mixture of MnCl_2_ and MPA was adjusted to specific pH by using NaOH solution (5 M) at anice-water bath. After aerating the mixture with N_2_ for 20 min, freshly prepared NaHSe solution was injected into the mixture under stirring. As-prepared MnSe nuclei solution was kept under N_2_ atmosphere in an ice-water bath. The molar ratio of Mn/Se is 0.048/1.0, and the optimal synthesis pH is 11.9.

Mn:ZnSe QDs were prepared by heating MnSe nuclei solution to 90 °C and following by gradual dropping of Zn stock solution that has the same pH with MnSe solution. Further heating for 3.5 h under N_2_ atmosphere resulted in Mn:ZnSe QDs. The final concentration of Se in the mixture is 0.83 mM and the feed ratio of Mn/Zn/Se/MPA is 1/62.5/20.8/160.

### Synthesis of Ag,Mn:ZnSe QDs

The synthesis process of Ag and Mn co-doped ZnSe QDs was totally similar to that of Mn doped ZnSe QDs. The mixture of AgNO_3_, MnCl_2_, and MPA was used to form the nuclei solution. The addition of Zn stock solution and followed by heating leads to the formation of Ag,Mn:ZnSe QDs. The total concentration of Se in the mixture is 0.83 mM. The optimized pH value is 11.9 and the optimized molar ratio of Mn/Ag/Zn/Se/MPA is 1/1.5/62.5/20.8/160.

## Additional Information

**How to cite this article**: Hu, Z. *et al.* Co-doping of Ag into Mn:ZnSe Quantum Dots: Giving Optical Filtering effect with Improved Monochromaticity. *Sci. Rep.* **5**, 14817; doi: 10.1038/srep14817 (2015).

## Supplementary Material

Supplementary Information
