# Supplementary material for: Co-doping of Ag into Mn:ZnSe Quantum Dots: Giving Optical Filtering effect with Improved Monochromaticity
Source: Sci Rep. 2015 Oct 8;5:14817. doi: 10.1038/srep14817 (PMC4597225; doi:10.1038/srep14817)
Supplement: Supplementary Information [file srep14817-s1.pdf]

Supporting Information:

Co-doping of Ag into Mn:ZnSe Quantum Dots: Giving Optical Filtering effect with Improved Monochromaticity

Zhiyang Hu, Shuhong Xu, Xiaojing Xu, Zhaochong Wang, Zhuyuan Wang, Chunlei Wang*, and Yiping Cui*

[*]Advanced Photonics Center, School of Electronic Science and Engineering, Southeast University, Nanjing, 210096 (P. R. China)

E-mail: wangchl@seu.edu.cn and cyp@seu.edu.cn

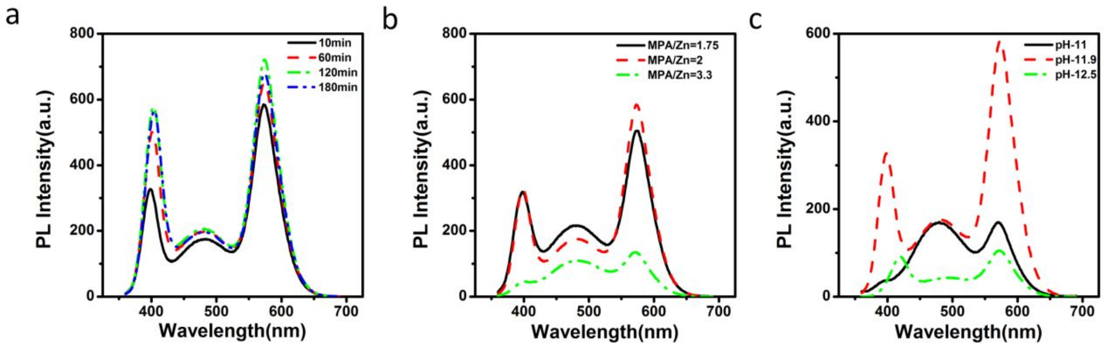

Fig. S1 | PL spectra of Mn:ZnSe QDs synthesized at different growth time (a), MPA/Zn ratios(b), and pH (c).

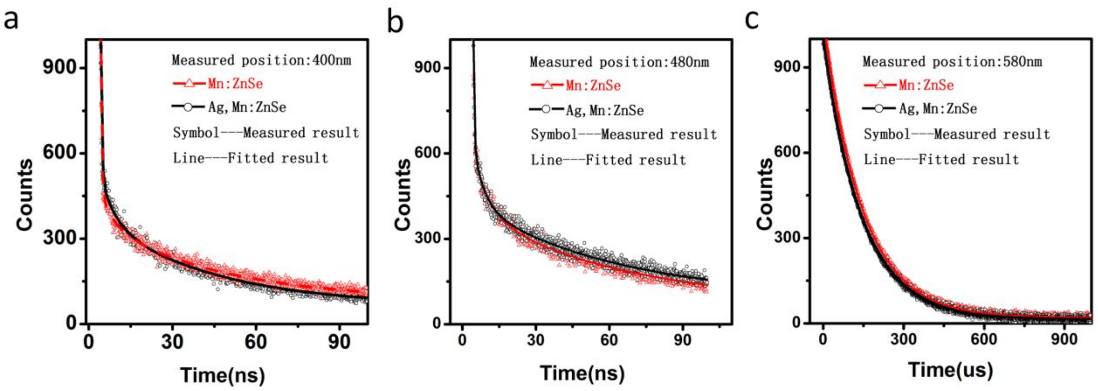

Fig. S2 | Time-resolved PL spectra. The measured PL decay spectra (symbols) and the fitted results (solid lines) of Mn:ZnSe QDs and Ag,Mn:ZnSe QDs for ZnSe band gap emission(a). and for ZnSe trap emission(b). and for Mn dopant emission(c).

According to the energy levels of defects, Mn and Ag dopants in Fig. 6, the PL lifetimes (symbolized by τ) and PL quantum yields (symbolized by QY) of QDs can be expressed by various irradiative decay rates (symbolized by k_r), non-irradiative decay rates (symbolized by k_{nr}), energy transfer rates (symbolized by k_{ET}), and the efficiency of the energy transfer (symbolized by Φ_{ET}).

Case 1：ZnSe band gap emission:

(I) For Mn:ZnSe QDs, the PL lifetimes and PL QY are expressed by[S1]:

$$\tau_{ZnSe} = 1/(k_r^{ZnSe} + k_{nr}^{ZnSe} + k_{ET}^{TS} + k_{ET}^{Mn}) \quad (1)$$

$$QY_{ZnSe} = k_r^{ZnSe} \times \tau_{ZnSe} \quad (2)$$

(II) For Ag,Mn:ZnSe QDs, the PL lifetimes and PL QY are expressed by:

$$\tau'_{ZnSe} = 1/(k_r^{'ZnSe} + k_{nr}^{'\,ZnSe} + k_{ET}^{'\,TS} + k_{ET}^{'\,Mn} + k_{ET}^{'\,Ag}) \quad (3)$$

$$QY'_{ZnSe} = k_r^{'ZnSe} \times \tau'_{ZnSe} \quad (4)$$

The PL QYs of ZnSe band gap emission can be measured from the emission of QDs in Fig. 1a, the ratio of PL QYs with and without Ag doping is about 16.The measured PL lifetimes are 37.4 ns for Mn:ZnSe QDs and 33.8 ns for Ag,Mn:ZnSe QDs as shown in Fig. S1 above. Therefore, according to equations 2 and 4 above, it can be safely deduced that the irradiative decay rates of ZnSe band gap emission for Mn:ZnSe QDs (k_r^{ZnSe}) is about 16 times than that of Ag,Mn:ZnSe QDs ($k'_r{}^{ZnSe}$). This result is comprehensible since the irradiative decay rate for band gap emission is relevant to the intrinsic properties of wavefunctions of the exciton, and the size, shape and the composition of QDs according to the reference viewpoint[S2]. After co-doping Ag inside Mn:ZnSe QDs, the alteration of QD composition and QD energy levels give rise to different values between k_r^{ZnSe} and $k'_r{}^{ZnSe}$.

Case 2：ZnSe trap emission:

(I) For Mn:ZnSe QDs, the PL lifetimes and PL QY are expressed by:

$$\tau_{TS} = 1/(k_r^{TS} + k_{nr}^{TS}) \quad (5)$$

$$\Phi_{TS} = k_r^{TS} \times \tau_{TS} \quad (6)$$

$$\Phi_{ET}^{TS} = k_{ET}^{TS} \times \tau_{ZnSe} \quad (7)$$

$$QY_{TS} = \Phi_{ET}^{TS} \times \Phi_{TS} \quad (8)$$

(II) For Ag,Mn:ZnSe QDs, the PL lifetimes and PL QY are expressed by:

$$\tau'_{TS} = 1/(k_r^{'TS} + k_{nr}^{'\,TS}) \quad (9)$$

$$\Phi'_{TS} = k_r^{'TS} \times \tau'_{TS} \quad (10)$$

$$\Phi'^{\,TS}_{ET} = k'^{\,TS}_{ET} \times \tau'_{ZnSe} \quad (11)$$

$$QY'_{TS} = \Phi'^{\,TS}_{ET} \times \Phi'_{TS} \quad (12)$$

In equation 5, a well known prerequisite for Mn:ZnSe QDs is the negligible energy transfer between the defects and Mn dopants are usually neglected due to the extremely low content of Mn inside QDs. Similarly, we can also neglect the energy transfer between the defects and Ag dopants in equation 9. That means the doping of Ag does not affect the irradiative decay rates of trap emission (namely $k_r^{TS}=k'^{TS}_r$). Since the measured PL lifetimes of trap emission for Mn:ZnSe QDs (51.4ns) and Ag,Mn:ZnSe QDs (54.2ns) are similar in Fig. S1 and the PL QYs of trap emission for Ag,Mn:ZnSe QDs is about 11 times lower than that for Mn:ZnSe QDs as can be measured in Fig. 1a.The PL quenching of ZnSe trap emission in equation 12 can be attributed to the lowered energy transfer efficiency (Φ_{ET}^{TS}) by contrasting equation 8 and 12.

Case 3：Mn dopant emission:

(I) For Mn:ZnSe QD,the PL lifetimes and PL QY are expressed by[S1]:

$$\tau_{Mn} = 1/(k_r^{Mn} + k_{nr}^{Mn}) \quad (13)$$

$$\Phi_{Mn} = k_r^{Mn} \times \tau_{Mn} \quad (14)$$

$$\Phi_{ET}^{Mn} = k_{ET}^{Mn} \times \tau_{ZnSe} \quad (15)$$

$$QY_{Mn} = \Phi_{ET}^{Mn} \times \Phi_{Mn} \quad (16)$$

(II) For Ag,Mn:ZnSe QDs, the PL lifetimes and PL QY are expressed by:

$$\tau'_{Mn} = 1/(k_r^{'Mn} + k_{nr}^{'Mn}) \quad (17)$$

$$\Phi'_{Mn} = k_r^{'Mn} \times \tau'_{Mn} \quad (18)$$

$$\Phi_{ET}^{'Mn} = k_{ET}^{'Mn} \times \tau'_{ZnSe} \quad (19)$$

$$QY'_{Mn} = \Phi_{ET}^{'Mn} \times \Phi'_{Mn} \quad (20)$$

According to the reference viewpoint[S2], the value of k_r^{Mn} is determined by the wavefunctions of d states of the Mn ion as well as the local environment around the Mn ion. From Figs.1a, 2, and S1, we can see nearly unchanged PL intensity, PL QYs, and PL lifetimes of Mn dopant emission before and after Ag doping (Ag/Mn no more than 1.5/1). It indicates Ag doping do not serious affect Mn emission at the default Ag doping amount (Ag/Mn of 1.5). Therefore, we think k_r^{Mn} is roughly equal to $k'_r{}^{Mn}$. According to equations 16 and 20 above, it means that the energy transfer efficiency for Ag,Mn:ZnSe QDs ($\Phi'_{ET}{}^{Mn}$) is also similar to that of Mn:ZnSe QDs (Φ_{ET}^{Mn}).

We estimate the errors for the ratio of k_r^{ZnSe}(Mn:ZnSe QDs) and $k_r^{'ZnSe}$(Ag,Mn:ZnSe QDs) with a quantitive manner. According to equations (2) and (4) in Supporting Information, the value of $k_r^{ZnSe}/k_r^{'ZnSe}$ is calculated by division of the value of QY_{ZnSe}/QY'_{ZnSe} via the value of τ_{ZnSe}/τ'_{ZnSe}. Therefore, the errors for $k_r^{ZnSe}/k_r^{'ZnSe}$ can be known via the errors of QY_{ZnSe}/QY'_{ZnSe} and τ_{ZnSe}/τ'_{ZnSe}. First, according to the reference reports[S3], the systematic error for PL QY measurement is about 5% by using dyes with the known PL QYs as a reference. Therefore, the value of QY_{ZnSe}/QY'_{ZnSe}=16±1.6. Though ZnSe band gap emission is low after Ag doping, the emission intensity is still within the detection scope of fluorescence spectrum. The signal to noise ratio is far more than 1000/1, giving neglectable error during PL emission measurement. Second, the errors for PL lifetime are also calculated. In this work, PL lifetimes for ZnSe band gap emission were fitted by biexponential equations. The error for each parameter are shown in Table. S1 below. The average lifetimes (τ_{avg}) are expressed by: $\tau_{avg}=(A_1\tau_1^2+A_2\tau_2^2)/(A_1\tau_1+A_2\tau_2)$, where τ_1, τ_2 are the time constants and A_1, A_2 are the amplitudes of the components. According this equation, the measured average lifetimes are 37.4±1 ns for Mn:ZnSe QDs and 33.8±0.9 ns for Ag,Mn:ZnSe QDs. Thus the value of τ_{ZnSe}/τ'_{ZnSe} range from 1.05 to 1.17. In conclusion, according to the range for the value of QY_{ZnSe}/QY'_{ZnSe} and the value of τ_{ZnSe}/τ'_{ZnSe}, we can get the the value of $k_r^{ZnSe}/k_r^{'ZnSe}$ range from 12.3-16.8. Obviously, even if we considered the errors during PL QY measurements and PL lifetime measurements, it is still no doubt that the doping of Ag giving rise to decreased k_r^{ZnSe}.

Sample	A_1	τ_1/ns	A_2	τ_2/ns	$\tau avg/ns$	R^2
Mn:ZnSe	491.3±66.4	3.4±0.3	305.3±3.6	41.8±1.7	37.4±1	0.97
Ag,Mn:ZnSe	559.3±53.8	3.8±0.3	346.0±5.2	38.6±1.5	33.8±0.9	0.98

Table. S1| Biexponential fitting parameters of PL lifetime for ZnSe band gap emission.

Sample	A_1	τ_1/ps	A_2	τ_2/ps	τ_{avg}/ps
Mn:ZnSe	0.00196	3.57	0.00217	39.57	22.5
Ag,Mn:ZnSe	0.00454	1.16	0.00267	26.47	10.5

Table. S2| Multi-exponential fitting parameters of ΔR/R data probed at 750 nm.

References

S1. Zheng, J.J. et al. Efficient Photoluminescence of Mn^{2+} Ions in MnS/ZnS Core/Shell Quantum Dots. *J. Phys. Chem. C* **113**, 16969-16974 (2009).

S2. Yang, Y.A., Chen, O., Angerhofer, A. & Cao, Y.C. Radial-Position-Controlled Doping of CdS/ZnS Core/Shell Nanocrystals: Surface Effects and Position-Dependent Properties. *Chem.Eur. J.* **15**, 3186-3197 (2009).

S3. Demas, J.N. & Crosby, G.A. Measurement of Photoluminescence Quantum Yield . Review. *J. Phys. Chem.* **75**, 991-1024 (1971).
